# CCTV Coverage Index Based on Surveillance Resolution and Its Evaluation Using 3D Spatial Analysis

**DOI:** 10.3390/s150923341

**Published:** 2015-09-16

**Authors:** Kyoungah Choi, Impyeong Lee

**Affiliations:** Lab. for Sensor & Modeling, Department of Geoinformatics, University of Seoul, Seoulsiripdaero 163, Dongdaemun-gu, Seoul 02504, Korea; E-Mail: shale@uos.ac.kr

**Keywords:** closed circuit television (CCTV), surveillance performance, surveillance coverage, surveillance resolution

## Abstract

We propose a novel approach to evaluating how effectively a closed circuit television (CCTV) system can monitor a targeted area. With 3D models of the target area and the camera parameters of the CCTV system, the approach produces *surveillance coverage index*, which is newly defined in this study as a quantitative measure for surveillance performance. This index indicates the proportion of the space being monitored with a sufficient resolution to the entire space of the target area. It is determined by computing *surveillance resolution* at every position and orientation, which indicates how closely a specific object can be monitored with a CCTV system. We present full mathematical derivation for the resolution, which depends on the location and orientation of the object as well as the geometric model of a camera. With the proposed approach, we quantitatively evaluated the surveillance coverage of a CCTV system in an underground parking area. Our evaluation process provided various quantitative-analysis results, compelling us to examine the design of the CCTV system prior to its installation and understand the surveillance capability of an existing CCTV system.

## 1. Introduction

CCTV surveillance operations have rapidly expanded due to the technology’s important role in crime prevention, traffic monitoring, and security [1]; although controversies regarding privacy and the effectiveness of CCTV installation have continually arisen [2,3]. Currently, many municipal governments throughout the world independently operate integrated CCTV control centers, whereby CCTV images are used to arrest criminals; additionally, corresponding news items can easily be encountered [4,5,6]. Furthermore, the use of CCTV in public locations such as shopping malls, apartments, and underground parking lots has reduced the possibility of crime, including theft, assault, and/or fraud [7,8,9,10]. The use of CCTV images has expanded beyond crime prevention; for example, to ensure the safety of people on a train-station platform; to observe public-transport passengers for unexpected behaviors; and to monitor patients at hospitals [11,12,13,14].

To solve problems regarding social welfare, transport safety, crime prevention, and other social issues, the establishment of CCTV systems in public and residential areas has been proposed [15]. Further CCTV systems were installed and existing systems were upgraded to decrease the blind spots, thereby improving the surveillance quality [16]. In addition to enhancing hardware specifications, the performance of CCTV systems can be improved by incorporating new software and technologies; for example, some researchers attempted to enhance the overall performance by optimizing the camera configuration in a CCTV system [17,18]. In addition, using GIS, they determine the optimal locations of CCTV following an analysis of CCTV images [19,20]. For example, a GIS tool, Isovist Analyst is used to identify a minimal number of CCTV for complete coverage of a target area based on a greedy search [21]. In reality, however, when analyzing CCTV performance in terms of quantifiable indicators, we usually calculate the ratio of the blind spots depending on whether or not the target areas are observable by a CCTV. The observable area is determined using the location and field of view of each camera on a two-dimensional (2D) ground plan of the target space. Importantly, this analysis may not provide sufficient accuracy because it does not consider three-dimensional (3D) locations and the distributions of the cameras, objects, and targets in the 3D space. For example, the coverage of a camera significantly differs according to the height of the camera and the target plane. Such a conventional 2D analysis may cause unnecessary overlapping coverage or over-estimated coverage. Further research is therefore required to provide a quantitative evaluation of surveillance performance including surveillance resolution or blind-spot calculations with the height in 3D space.

With the improved Building Information Modeling (BIM) technology, both the physical and functional characteristics of a building can now be generated in a digital format [22]. As a result, the creation, visualization, and simulation of a 3D virtual model of a building can be performed more conveniently [23,24]. The BIM models allow for the manipulation of surveillance locations and viewpoints; therefore, the idea of using the BIM as the basis for simulating CCTV coverage has been proposed and verified to determine surveillance performance [25,26]. By referring to this idea, the redundant overlapping coverage of CCTV could be effectively prevented with the generation of a 3D model during the design phase of a CCTV system; however, it will still not be possible to determine the quality of the surveillance performance using the resolution at which an object can be identified in a given area. For example, the use of CCTV images to trace the movements of a suspect at a crime scene may not provide an image of the suspect’s face; or even if it does, the resolution is insufficient for facial recognition. This may occur because the surface direction of the target object was not considered and the achievable resolution was not computed through a simulation process. Although the target area appears covered at the ground level, the coverage of each camera narrows as the height increases from the ground. As most targets are off the ground, the surveillance performance for the target is lower than the results from the existing evaluation method, which calculates coverage at the ground level. Additionally, the existing evaluation method assumes that the target is facing toward the camera; however, targets are usually looking in a horizontal direction so that the surveillance performance is significantly reduced when compared with the existing method.

**Figure 1:**
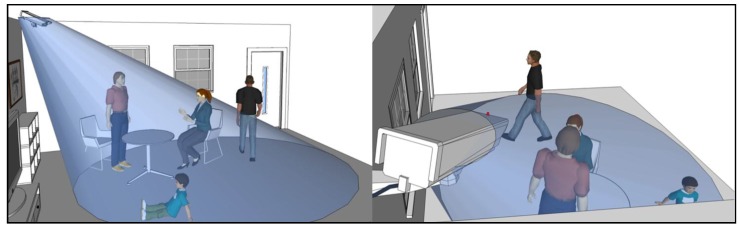
It is not possible to observe the faces of the four people from the image even though they exist in the 2D coverage of a CCTV camera.

In Figure 1, the weak points of the existing surveillance-performance evaluation method are shown, whereby there are four people in the 2D coverage of a CCTV camera; therefore, complete surveillance is achieved for the four people based on the existing method. The faces of the people, however, cannot be recognized. Three-dimensional coverage of the face of the male in the black T-shirt is not observable from the camera image, while the entire face of the female in the green jacket is hidden by the male in the red polo shirt. The male in the red shirt is positioned with his back to the camera so that his face cannot be detected from the camera image. The child is looking at the TV in a different direction from the optical axis of the camera and is sitting in a lower position away from the optical axis of the camera; consequently, the resolution of the camera image is insufficient to recognize his face even though his face is in the 3D coverage. With commercial software such as VideoCAD, we can check 3D coverage and simulate images on virtual avatars captured by a certain camera in a site interactively through a 3D graphic interface. However, because the software does not provide any comprehensive quantitative indicator about surveillance performance of the system, we still cannot understand how completely a CCTV system monitors the target area in a certain level of detail [27].

In this study, we therefore propose new comprehensive indicators to quantify CCTV-surveillance performance, which quantitatively represents how completely a target space can be monitored by a CCTV system with a degree of detail enough for given surveillance requirements. Here, it is carefully considered that the details of objects appearing in a CCTV image depend on types and specifications of the sensors in the system and the orientation as well as location of the objects in the target area. We also develop a performance evaluation method using the proposed indicators and applied it to a real case to verify its feasibility. The remaining part of this paper is organized as follows: proposed concepts and methods are explained in Section 2; examples of surveillance coverage evaluation are shown in Section 3; and conclusions are presented in Section 4.

## 2. Surveillance Coverage Evaluation

Surveillance performance indicates how effectively a target area is being monitored by a CCTV system. As quantitative measures of surveillance performance, we propose the following two indicators: surveillance resolution and surveillance coverage index. Surveillance resolution indicates how closely a specific object can be monitored with a CCTV system, depending on the location and orientation of the object as well as the cameras of the CCTV system. Surveillance coverage index focuses on a specific region rather than an object, indicating how completely a region of interest can be monitored with more than a specified surveillance resolution. The region can also be a path, an area, or a 3D space as a subset of the object space; for example, to what extent a pedestrian path in a parking lot, a crowded area in a mall, or the entire inner space of a building can be completely monitored may be of interest. The resolution threshold can be established according to its own application; for example, it can be two px/cm for facial recognition.

The evaluation process to derive the proposed performance indicators requires two kinds of inputs. The first group includes those with almost constant properties—at least during the evaluation process—which are a 3D geometric model of the object space, and the intrinsic and extrinsic parameters of all of the cameras of the CCTV system. The second group includes the changeable parameters, which are the resolution threshold and the regions of interest that are specified within the object space.

In this section, we first describe the definition and derivation of the proposed indicators, the surveillance resolution of an object with a specified location and orientation, and the surveillance coverage index for a specified region of interest. We then explain the proposed evaluation process to derive these indicators in an actual practical situation.

### 2.1. Surveillance Resolution and Coverage Index 

The following four types of resolutions are used to define the quality of an image: geometric, radiometric, spectral, and temporal. Among these resolutions, the geometric resolution is the most effective when describing an object’s geometric properties such as position and shape. The geometric resolution is typically expressed in terms of Ground Sampling Distance (GSD), which refers to the distance of an object surface in a single pixel of an image. In this context, the surveillance performance of a CCTV can be evaluated by observing the minimum GSD required to monitor an object. An arbitrary length can therefore be set within the target area in the object space and the actual length projected on the CCTV image can be calculated; we define the latter value as the surveillance resolution and apply it when assessing the surveillance performance of a CCTV. The surveillance resolution depends on sensor’s physical characteristics, such as the focal length, the principle point, the pixel size, the projection type. It also varies according to the relative geometric relationship between the camera and the object. Even with an object at the same position, the resolution can be different in accordance with the orientation of the object surface. By considering these diverse factors affecting the resolution, we derive a formula to derive the resolution as follows.

**Figure 2:**
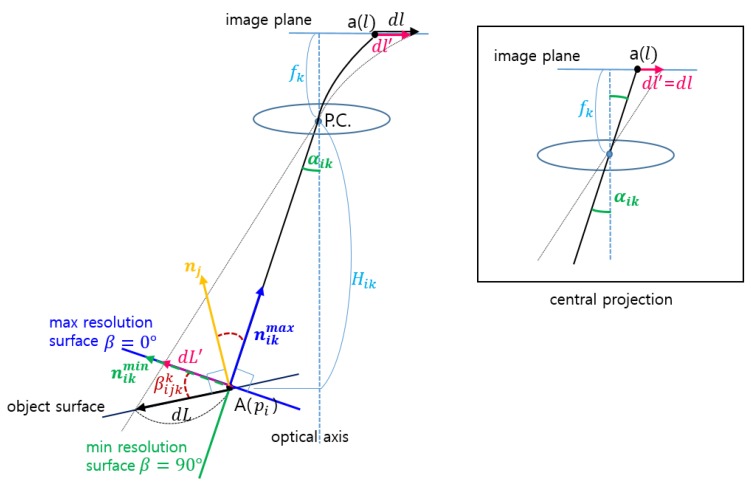
Definition of surveillance resolution.

When an object locating at a location with its surface normal is monitored by a CCTV camera, it is projected to the image with a resolution. The defined surveillance resolution is represented as the ratio between the actual length (dL) of an object and its projected length (dl′) on the image, which is defined as Equation (1). It consists of four terms that accurately model four steps of the projection process from the object space to the image space. The steps are computing (1) the projected length $\left(dL^{\prime }\right)$ of dL on the surface where the object can be observed at a maximum resolution; (2) the incident angles (α) when the object is projected through the perspective center; (3) the projected length (dl) of dL on the image according to a lens formula without any distortion; and (4) the projected length (dl′) of dL on the image considering distortions, respectively. Figure 2 illustrates the geometric meanings of the main parameters associated with the derivation of the defined surveillance resolution.

(1) $$r\equiv \frac{dl\prime }{dL}\equiv \frac{dL\prime }{dL}\cdot \frac{d\alpha }{dL\prime }\cdot \frac{dl}{d\alpha }\cdot \frac{dl\prime }{dl}$$

The first term considers the orientation of the object surface. In spite of the same object position, the resolution of its projection can be different from the object’s orientation. The highest resolution can be achieved when the surface normal corresponds to the direction toward the perspective center. The length projected to the surface and directed to the perspective center can be derived as the following:

(2) dL′=dLcosβ

where β is the angle between the actual orientation and the orientation resulting in the highest surveillance resolution.

The second term transforms the projected length $\left(dL^{\prime }\right)$ into the range of the incident angle (α), which can be derived based on the arc length formula, as follows:

(3) $$d\alpha =\frac{dL\prime }{D}=\frac{dL\prime }{H}\text{cos}\alpha$$

where D is the distance from the object to the perspective center; and H is its projected length to the optical axis, which is represented as Dcosα.

The third term reflects a projection model. In a narrow angle system, it is usually assumed that the central projection model is available, whereas different models apply for wide angle systems, which are mostly utilized for CCTV systems. Some useful models are presented as Equation (4). Through a lens of a focal length (f), an object point in the direction (α) is projected to a position (l) distant from the principal point. The first of the models in Equation (4) signifies the central projection model, while the last term models the distortion correction. If we consider only the radial distortion of the lens, the correction model can be expressed as Equation (5).

(4) $$l=\;\{\begin{array}{c} f\text{tan}\alpha \\ 2f\text{tan}\frac{\alpha }{2}\\ f\alpha \\ 2f\text{sin}\frac{\alpha }{2}\\ f\text{sin}\alpha \end{array}dl=\;\{\begin{array}{c} f{\text{sec}}^{2}\alpha d\alpha \\ f{\text{sec}}^{2}\frac{\alpha }{2}d\alpha \\ fd\alpha \\ f\text{cos}\frac{\alpha }{2}d\alpha \\ f\text{cos}\alpha d\alpha \end{array}$$

(5) $$l^{\prime }=\;l_{0}+\;l+k_{1}l^{3}+k_{2}l^{5}\;dl^{\prime }=\;\left(1+3k_{1}l^{2}+5k_{2}l^{4}\right)dl$$

As shown in Figure 2, when an object (A) locating at location $\left(p_{i}\right)$ with its surface normal $\left(n_{j}\right)$ is monitored by a CCTV camera $\left(c_{k}\right)$, it is projected to a CCTV image with a resolution $\left(r_{ijk}\right)$. The defined surveillance resolution is represented as the ratio between the actual length (dL) of an object (A) and its projected length (dl), which is formulated as Equation (6). Here, it is assumed that the camera follows the central projection without any distortion:

(6) $$r_{ijk}=f\left(p_{i},n_{j},c_{k}\right)=\frac{dl}{dL}=\frac{\text{cos}(\beta_{ijk})}{dL\prime }\frac{dL\prime }{\text{cos}(\alpha_{ik})}\;\frac{f_{k}}{H_{ik}}=\frac{f_{k}}{H_{ik}}\frac{\text{cos}(\beta_{ijk})}{\text{cos}(\alpha_{ik})}$$

The vertical distance $\left(H_{ik}\right)$ between the object and center of projection and the offset angle from the optical axis $\left(\alpha_{ik}\right)$ are determined based on A’s location $\left(p_{i}\right)$, and the camera’s position and attitude. According to the orientation $\left(n_{j}\right)$, the angle $\left(\beta_{ijk}\right)$ between the actual orientation of A $\left(n_{j}\right)$ and the orientation resulting in the highest surveillance resolution $\left(n_{ik}^{max}\right)$ is decided. The $n_{j}$ and $n_{ik}^{max}$ represent the normal vector of A on the object surface and the normal vector of A on the image plane at the maximum surveillance resolution possible, respectively. In addition, focal length $\left(f_{k}\right)$ is computed from the camera modeling process.

Although CCTV cameras will acquire images at a maximum resolution of A if the object orientation is facing $n_{ik}^{max}$, the resulting image resolution will be lower when the object is placed on the surface tilted by $\beta_{ijk}$. When the angle of $\beta_{ijk}$ increases to 90°, the object will not be identifiable in the images. Moreover, the surveillance resolution will have a negative value when $\beta_{ijk}$ is larger than 90°, and this is the case when the opposite side of the object is projected to the image; for example, only the back of a suspect is captured in the CCTV image when the intention was to observe the facial features of the suspect. The negative surveillance-resolution value is not useful information in this case and it is therefore replaced by 0.

The camera parameters describe its projection characteristics. The intrinsic parameters are focal length, principal point, and distortion coefficients, whereas the extrinsic parameters are the position and orientation of the camera in an object coordinate system. As the camera parameters can be estimated through a camera modeling process, such as self-calibration using the acquired CCTV images and reference data, the intrinsic and extrinsic parameters can be assumed as known. In addition, the same camera parameters can be applied in cases where the coverage originates from an identical camera. The surveillance resolution $\left(r_{ijk}\right)$ of a camera is therefore derived from an object in a specific location and orientation in a target space.

Referring back to Equation (6), $\alpha_{ik},\beta_{ijk},$ and $H_{ik}$ must be known to determine the surveillance resolution at a certain location and orientation. As it is assumed that the CCTV camera’s intrinsic and extrinsic parameters and the object’s location and orientation are known, the following equations can be used to compute $\alpha_{ik},\beta_{ijk},$ and $H_{ik}$. First, the distance from the object location $p_{i}\left(X_{pi},Y_{pi},Z_{pi}\right)$ to the center of the projection $\left(D_{ik}\right),$ and the unit vector of the optical axis $\left(v_{ck}\right)$ in a 3D coordinate system defined by the camera’s extrinsic parameters $\left(X_{ck},Y_{ck},Z_{ck},\omega_{ck},\phi_{ck},\kappa_{ck}\right)$ are calculated. Then, the normal vector of the surface where the CCTV camera can monitor at maximum resolution $n_{ik}^{max}$ can be calculated using Equation (7), as follows:

(7) $$n_{ik}^{max}=\frac{1}{D_{ik}}\left[X_{ck}-X_{pi},\;Y_{ck}-Y_{pi},Z_{ck}-Z_{pi}\right]$$

Next, the off-axis angle from the CCTV camera’s optical axis $\alpha_{ik}$ is determined by Equation (8) and Equation (9) is used to compute $H_{ik}$. In addition, the angle created by the actual surface where the object exists, and the surface at which the CCTV camera can observe the object at maximum resolution $\beta_{ijk}$ can be calculated with the relation shown in Equation (10), as follows:

(8) $$\text{cos}\left(\alpha_{ik}\right)=n_{ik}^{max}\cdot v_{ck}$$

(9) $$H_{ik}=D_{ik}\text{cos}\left(\alpha_{ik}\right)$$

(10) $$\text{cos}\left(\beta_{ijk}\right)=n_{ik}^{max}\cdot n_{j}$$

The values of $\alpha_{ik},\beta_{ijk},H_{ik}$ vary with respect to the local reference frame defined by the CCTV camera and the object as expressed in Equations (8)–(10), and these values will determine the surveillance resolution defined by Equation (6). Nonetheless, in general, multiple cameras are installed over the target area and one object may appear in many camera images. As the relative position and orientation differ between each CCTV camera and the particular object, each image will attain different surveillance resolutions. Although there may be different surveillance resolutions for a given object, the largest value will be assigned as the object’s surveillance resolution. Consequently, the surveillance resolution of an object in a CCTV system with multiple cameras $\left(r_{ij}\right)$ can be expressed as the following, where l is the total number of cameras in a CCTV system:

(11) $$r_{ij}=\text{max}\left(r_{ijk}|1\leq k\leq l\right)$$

The surveillance resolution $r_{ij}$ is calculated with $\alpha_{ik},\beta_{ijk},H_{ik}$, which are obtained with respect to the object’s position and orientation in a space. To assess the surveillance coverage (η), the surveillance resolution at every possible position and orientation in a given space is produced. Then, the percentage of the surveillance resolution that exceeds over a pre-defined threshold can be computed by Equation (12), as follows:

(12) $$\eta =\frac{N\left(r_{ij}\geq r_{th}\right)}{n\times m}$$

where n is the total number of positions sampled; m is the total number of orientations sampled at each position; and N( ) is the number of samples that meet the requirement in the bracket.

### 2.2. Coverage Evaluation Procedure

**Figure 3:**
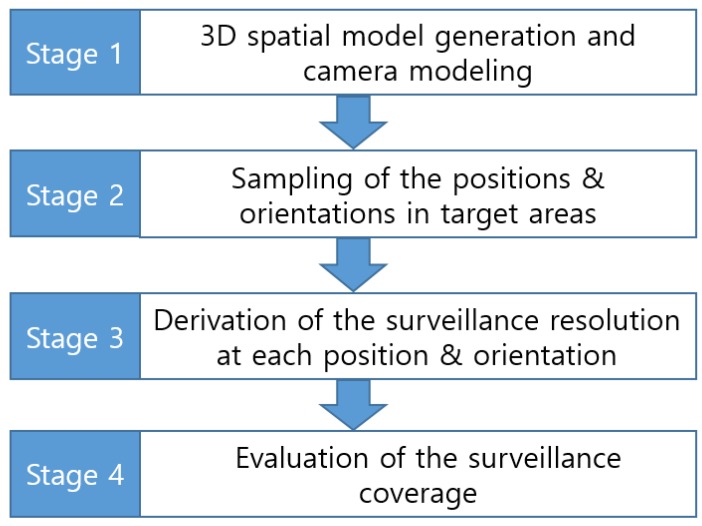
Surveillance Coverage Evaluation Procedure.

By applying the surveillance resolution and coverage index described in Section 2.1, the evaluation of surveillance coverage can be conducted using the steps shown in Figure 3. First, we need to generate 3D spatial models of the target surveillance area and determine the CCTV camera’s extrinsic and intrinsic parameters. Next, samples are selected at each location, whereby the object is visible from different orientations. Then, the surveillance resolutions at each of the sampled locations and orientations are derived from Equation (1). Finally, the completeness of the surveillance coverage is evaluated by computing the surveillance coverage index based on Equation (12).

In the first stage, we need to generate polyhedral models of the area from existing 2D architectural floor plans or newly acquired sensory data. If we use the floor plans, rather than using other sensory data, it is easier to create the corresponding 3D model of the building; however, floor plans contain the detailed architectural design of a building, and inconsistencies between the designed model and the actual building construction may exist. To accurately model the physical building, we need to acquire the sensory data of the area, such as images and laser-scanning data; then, the polyhedral model can be generated manually from stereo images or semi-automatically from point clouds. With the 3D polyhedral model, we need to know the camera’s extrinsic and intrinsic parameters. For extrinsic parameters, we have the location of the camera’s perspective center, expressed by three coordinates, and its attitude, expressed by three independent rotation angles. To obtain the parameter values, we distribute GCPs (Ground Control Point) in the area and perform bundle adjustment with images including the GCPs. The intrinsic parameters describe the metric characteristics of a camera, and can be determined through a camera-calibration process using a specially designed calibration target. The position of principal point, focal length, distortion amount, and pixel size are the main intrinsic parameters. In this case, unlike most, we cannot change the camera’s position and attitude, so we must position the calibration target on the floor in various directions and acquire images of the target.

In the second stage, we determine the amount of sampling desired from all of the possible 3D locations of the object, along with their corresponding orientations, in the entire target space. For the determination of sampling locations, we first define the target space in an arbitrary 3D Cartesian coordinate system. Then, each of the axes is divided to form a 3D grid. It is possible to define the surveillance resolution at each of the 3D grid points by using $p_{i}=\left\{p_{1},p_{2},\ldots ,p_{n}\right\}$; for instance, when the three axes are split every 10 cm in a $1\;\times \;1\;\times \;1{\text{ m}}^{3}$ space, there would be a total of 1000 locations to sample. On the other hand, the orientations that an object can face range from 0° to 360° horizontally, 0° to 180° vertically, and 0 π to 4 π for their solid angle. To include all of the possible orientations, we divide the angles both horizontally and vertically using an arbitrary location as the center. Using the same idea that applied to the 3D grid points, the surveillance resolution of all of the orientations at a given location can be found by using $n_{j}=\left\{n_{1},n_{2},\ldots ,n_{m}\right\}$; for example, if the sampling was conducted at an interval of 1°, the total orientation samples for a given location will be 360 × 180=64,800. When the interval is increased to 10° or 45° to reduce the number of orientation samples, there would be 648 and 32 orientations, respectively. This means that even when 8 × 4=32 orientations are observed at each location, the number of calculations for finding the surveillance resolution reach 32,000 in a 1 m^3^ with 1000 locations.

The advantage of using such a method to sample at every given interval for both location and orientation seems logical; however, the disadvantage is that each of the defined orientations do not cover the same solid angle, whereby the solid angle covered by each orientation decreases as the vertical angle increases. The vertices of the regular polyhedrons inscribed in a sphere or the center of their faces therefore provide the same solid angle across the orientations for a location. The five regular polyhedrons are the tetrahedron, hexahedron, octahedron, dodecahedron, and icosahedron. For example, assuming a particular location as the center of an icosahedron, orientation sampling can be made facing each of the vertices, which will provide the same solid angle throughout and the number of samples will decrease to 12.

In the third stage, when deriving surveillance resolutions at each of the sampled locations and orientations, we must analyze the visibility of the position from a camera by applying a ray-tracing algorithm. With this algorithm, we define a ray from the position to the perspective center of the camera and determine whether this line is intersected with other obstacles. If an intersection is determined, the position is within the occluded area of the camera and we cannot define the surveillance resolutions at the position. Before applying ray-tracing for each position, we need to compute the horizontal coverage of every camera and the 2D Minimum Bounding Rectangle (MBR) of all of the obstacles in the target area. Although the obstacles are defined in a 3D sense, most of them are extended to the ceiling starting from the ground, with the same horizontal outline such as a pillar. In this case, by examining the 2D overlap, we can determine whether it is overlapped in a 3D sense without a complicated 3D process. 

Ray-tracing for the calculation of the surveillance resolution at a position with an orientation by a camera is performed as follows: (1) determine whether the position is within the horizontal coverage of the camera; (2) determine the 2D MBRs of the line between an object point (a sampled position) and the perspective center; (3) check whether there is a 2D overlap between the MBRs of a line and an obstacle; if there is no 2D overlap, stop at this step with no 3D overlap; (4) check whether the line segment intersects with the 2D boundary polygon of the obstacle; if it does, stop at this step because the obstacle has an identical horizontal outline according to the height, signifying that it also intersects with the obstacle in a 3D sense; (5) determine if the line segment intersect with a 3D polyhedron model of the obstacle; and (6) if there is an overlap, the position cannot be identified in the camera image and the surveillance resolution is set to 0.

In the final stage, we compute the surveillance coverage index by comparing the achievable resolution with the desired one. Here, we determine the proportion of the instances that reach above the desired resolution among the resolution values computed at all the sampled locations and orientations in the previous stage. The desired resolution can be established or derived from the specified surveillance requirements for recognition and tracking. For example, one may want to monitor every location in the target space with a resolution of 0.5 px/cm required for a meaningful recognition process. Using the resolution values computed in the previous stage, we can easily compute the proportion of the locations monitored with at least the desired resolution and present it as the overall coverage index of the target space. In addition, we can visualize the computed surveillance resolution at each location with different object orientations in the 2D/3D space to visually inspect the weak and strong surveillance areas. Furthermore, we can check the coverage index for a special surveillance sector such as a doorway or moving path in a parking area. In a doorway, one may want to recognize even the faces of the people going in and out through an exit; and for the face recognition, the required resolution of at least 2 px/cm may be assumed. We can also determine how well such a requirement is satisfied in the space of interests in a quantitative way by checking the computed resolution values at all the locations with different orientations within the target space. In addition, we can identify the weak area and propose the location and orientation for additional camera installation to fulfill surveillance requirements. Adding cameras or constructing a CCTV system involves a major expense; therefore, we need an elaborate design for achieving the surveillance purpose before the installation. In addition, there are available so many different cameras with different prices and performance, and thus we can check the surveillance performance by changing the camera models or the camera parameters to derive more optimal camera specifications and configurations.

## 3. Application Example and Analysis

### 3.1. Experimental Data

**Figure 4:**
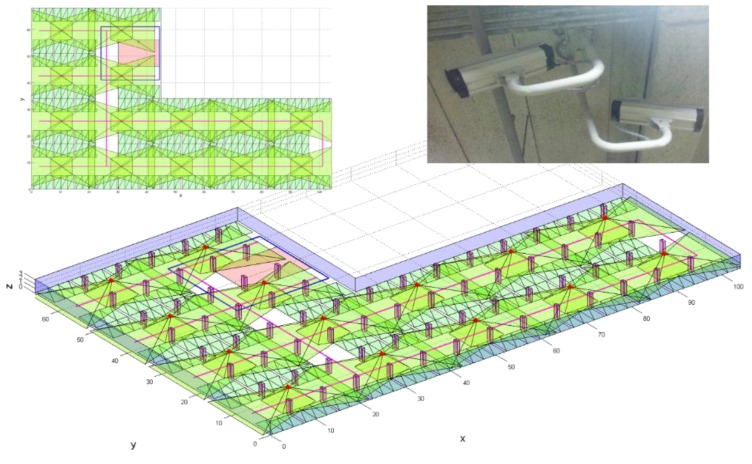
Arrangement of CCTV cameras in an underground parking lot.

The underground parking lots of buildings such as apartments are the places where CCTV systems are encountered in daily life. We therefore produced a simulation of a typical configuration and size of a parking lot, like that in Figure 4, based on the concept of the proposed surveillance resolution for the evaluation of the surveillance coverage of the target area. The surface area of the generated parking lot is 5079.47 m^2^ with a height of 3 m. Additionally, the CCTV cameras were positioned to reflect the real world, whereby the cameras were installed in pairs, facing the opposite direction. In addition, CCTV cameras are usually installed on the ceiling of the path that the cars and people mostly use. Here, we assumed that each pair of cameras is rotated by ±12° in the y-axis, and the approximate distances between them are 20 m in the x-axis and 17 m in the y-axis. We also assumed that the focal length, pixel size, and detector dimension of each camera are 10 mm, 5 µm, and 4000 by 3000 pixels, respectively. In this case, the coverage of each camera is about 6 m by 4.5 m at a place of 3 m distance.

### 3.2. Evaluation Results and Analysis

#### 3.2.1. Overall Analysis

**Figure 5:**
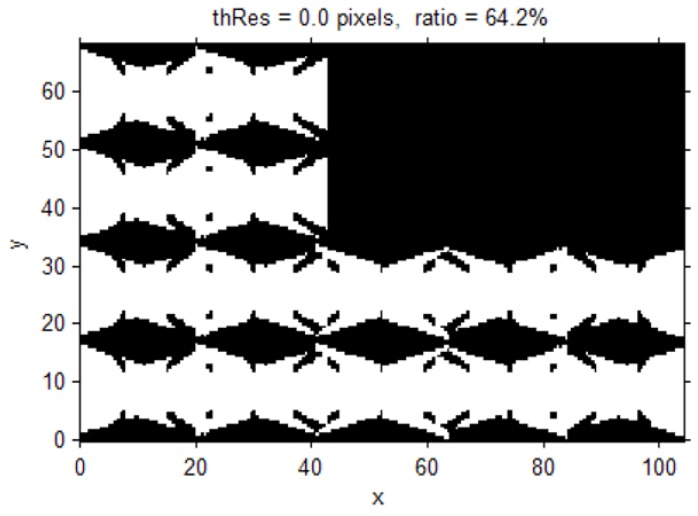
Areas with a surveillance resolution greater than 0 when objects are on the bottom and their orientations are upper vertical (white: resolution > 0 px/cm; black: resolution = 0 px/cm).

The possible locations of the object were sampled at each of the 3D grid points with an interval of 50 cm and the possible orientations of the object were sampled every 90° in the horizontal and vertical planes. Then, the observed surveillance resolutions from the CCTV cameras were determined for all of the samples. In Figure 5, the horizontal position of the object monitored above a surveillance resolution of 0 is displayed, when the object is located on the ground (z=0 m) and is facing the ceiling. Out of the total 20,541 ground locations, 13,186 locations can be observed in CCTV images, and 64.2% of the ground surface is computed as the surveillance area. This evaluation of surveillance coverage is similar to an existing method for determining blind spots, whereby the coverage of CCTV cameras in 2D space is derived [17,28].

**Figure 6:**
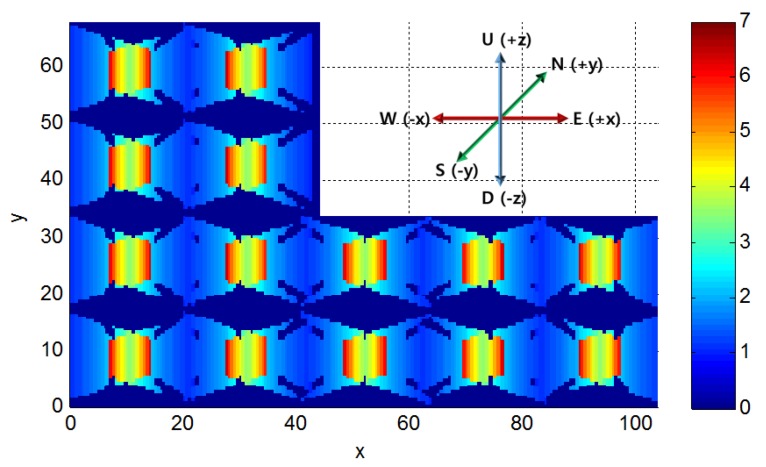
Surveillance resolutions when objects are on the ground and their orientations are upper vertical (blue: resolution = 0 px/cm; red: resolution = 7 px/cm).

**Figure 7:**
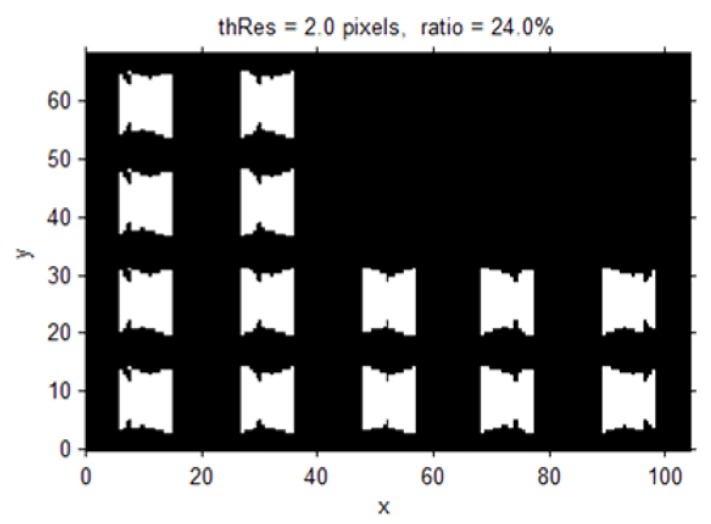
Areas with a surveillance resolution greater than or equal to 2 px/cm when objects are on the bottom and their orientations are upper vertical (white: resolution ≥ 2 px/cm; black: resolution < 2 px/cm).

For example, to identify a suspect who has trespassed through a parking lot, the suspect should appear in a CCTV image and his face should be recognizable from the image; however, it is difficult to estimate the success of such an objective when the surveillance coverage is determined with the existing 2D blind-spot-analysis method. Although slight parameter differences exist between CCTV cameras, the resolution that is typically required for monitoring an object in detail is about 2 px/cm, whereas it is about 0.7 px/cm for general surveillance (Theia Technologies, 2009). Accordingly, the surveillance resolution must be at least 2 px/cm to distinguish the appearance of a suspect without a criminal record. The surveillance resolution from the ground surface when the orientation of an object is upper vertical is illustrated in Figure 6; accordingly, a surveillance resolution exceeding over 2 px/cm is displayed in Figure 7. The resulting outcome produced 4930 locations, from the total of 20,541, as the positions that exceeded over 2 px/cm, signifying that, at this height, the suspect’s facial features can be identified approximately 24% of the time. With a CCTV-surveillance-coverage evaluation method like that which is previously explained, the percentage of surveillance resolutions that reach the required resolution to successfully fulfill the CCTV system’s purpose can be determined.

**Figure 8:**
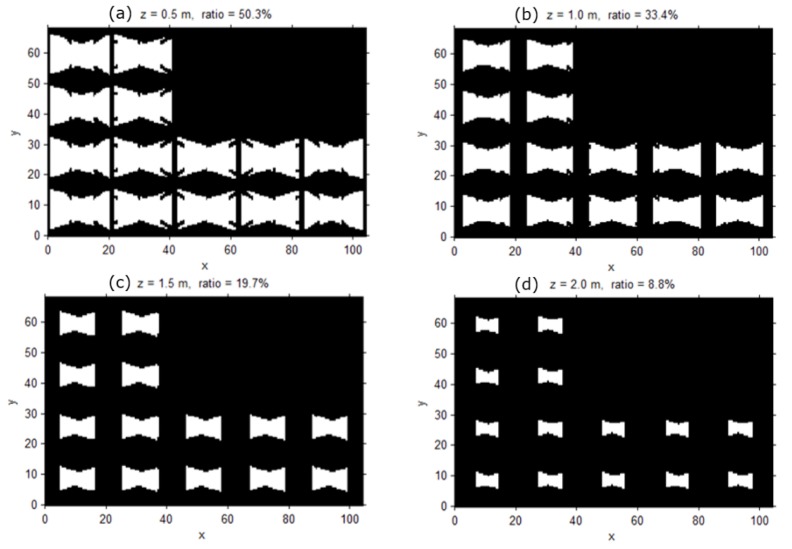
Surveillance coverage index with a threshold of 1 px/cm according to the elevation when objects have an upper-vertical orientation; (**a**) the index at the elevation of 0.5 m; (**b**) the index at the elevation of 1 m; (**c**) the index at the elevation of 1.5 m; (**d**) the index at the elevation of 2 m.

As the height of the object increases, the surveillance coverage significantly decreases, as shown in Figure 8; this represents a surveillance coverage index with a threshold of 1 px/cm according to the elevation when objects have an upper-vertical orientation. Figure 8c shows that the subject’s face could be observed in the area with a probability of 19.7% if the individual’s face is at a 1.5 m height and looking in an upper-vertical direction.

**Figure 9:**
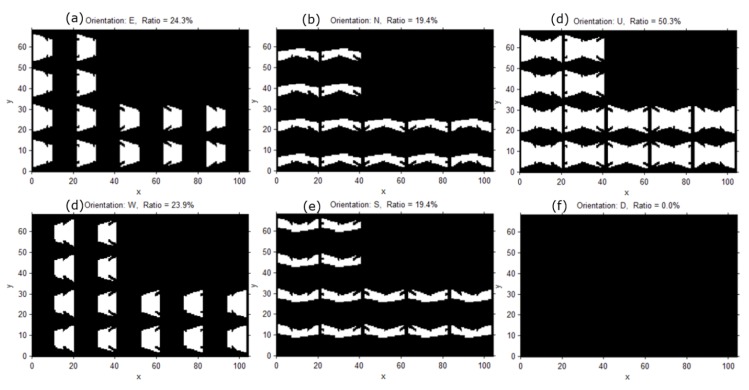
Surveillance coverage index with a threshold of 1 px/cm according to the orientation when objects are at an elevation of 0.5 m; (**a**) the index with the eastward orientation; (**b**) the index with the northward orientation; (**c**) the index with the upward orientation; (**d**) the index with the westward orientation; (**e**) the index with the southward orientation; (**f**) the index with the downward orientation.

Furthermore, the surveillance coverage index changes according to the object’s orientation. Figure 9 shows the surveillance coverage index with a threshold of 1 px/cm according to the orientation when objects are at an elevation of 0.5 m; 0.5 m is the general height of a car’s license plate. Figure 9f shows that if an object is moving in the area looking downward, it is not possible to recognize the object’s surface.

#### 3.2.2. Areal Analysis

**Figure 10:**
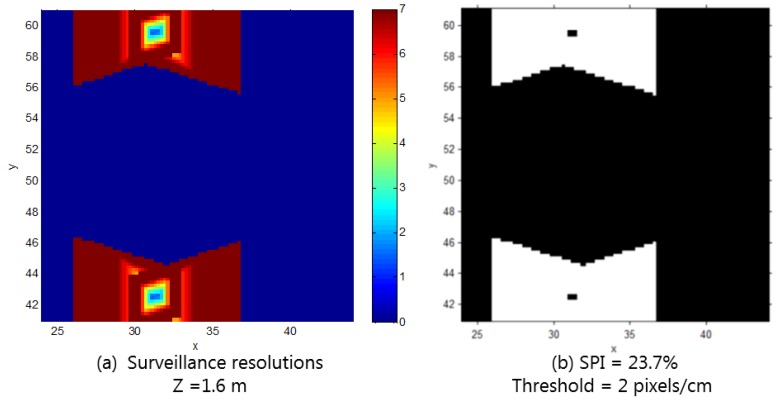
(**a**) Surveillance resolutions when objects are at a 1.6 m height and their orientation is in the direction in which the maximum resolution is achieved; (**b**) Surveillance coverage index with the threshold of 2 px/cm.

The suggested methodology incorporates the geometric properties and movement trend of an object to allow for a detailed evaluation of the surveillance coverage in a 3D space; for example, the red rectangular area in Figure 4 represents the entrance from a parking lot into a building, such as an escalator or elevator, where more careful monitoring is required. Surveillance coverage in such areas can therefore be determined to analyze the vulnerability of the CCTV system when it comes to crime prevention and reaction. For this analysis, we limited the vertical range—from 1.2 m to 1.8 m—in consideration of the average height of a human face, and sampled the target space of this vertical range with locations of 20 cm intervals, which is the space denoted with the blue line in Figure 4. At each location, we considered four horizontal orientations with a 90° interval. The number of sampling of locations and orientations in the area totaled 100(x) × 100(y) × 4(z) × 4 (horizontal angle) × 1 (vertical angle) = 160,000, where all of their surveillance resolutions were determined. The head of a person would roughly be at a height of 1.6 m, when the average height of adults is taken into account. Illustrations of the surveillance resolutions at the 1.6 m height when the orientation is in the direction in which the maximum resolution is achieved are shown in Figure 10. The corresponding surveillance coverage index is 23.7%, and it can be concluded that it is very difficult to verify the identity of an individual in the target area using the CCTV system.

**Figure 11:**
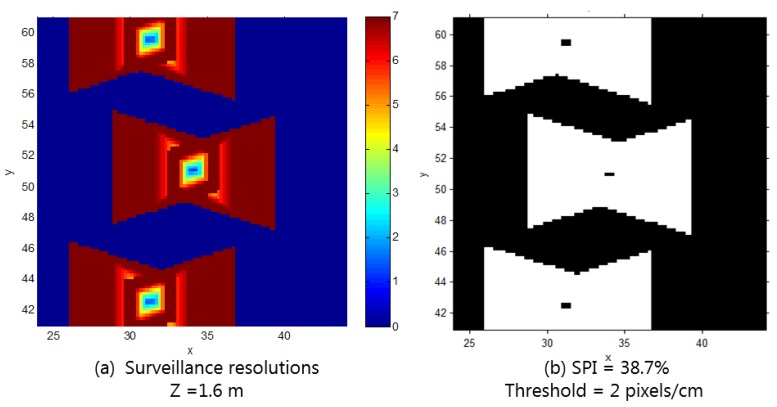
(**a**) Surveillance resolutions when objects are at 1.6 m height and their orientations are in the direction in which the maximum resolution is achieved; (**b**) Surveillance coverage index with a threshold of 2 px/cm (after adding two cameras).

To improve the existing monitoring quality, the installation of two additional CCTV cameras is planned. We examined the difference of the target-area resolution after the additional cameras are installed, with a possible location as the center (34, 51, 3) and a rotation of ±12° in the y-axis. Figure 11 shows the surveillance resolutions at the height of 1.6 m, when the orientation is in the direction in which the maximum resolution is achieved, after the cameras are added. In this case, we can conclude that, by adding the additional cameras at the possible location, the surveillance coverage for the target area was enhanced, as the surveillance coverage index increased from 23.7% to 38.7%.

Table 1 shows the surveillance coverage index at different elevations when an object is facing the direction that provides the maximum resolution, before and after adding the cameras. From this, we can check that the surveillance coverage for the target area is improved by approximately 60% after the two cameras are added; however, as the height of the object increases, the surveillance coverage significantly decreases.

**Table 1:** Surveillance coverage index before and after adding cameras (unit: %).

	Z = 1.2 m	Z = 1.4 m	Z = 1.6 m	Z = 1.8 m	Total
Before	35.0	28.9	23.7	18.4	26.5
After	59.9	48.5	38.7	29.9	44.3

**Figure 12:**
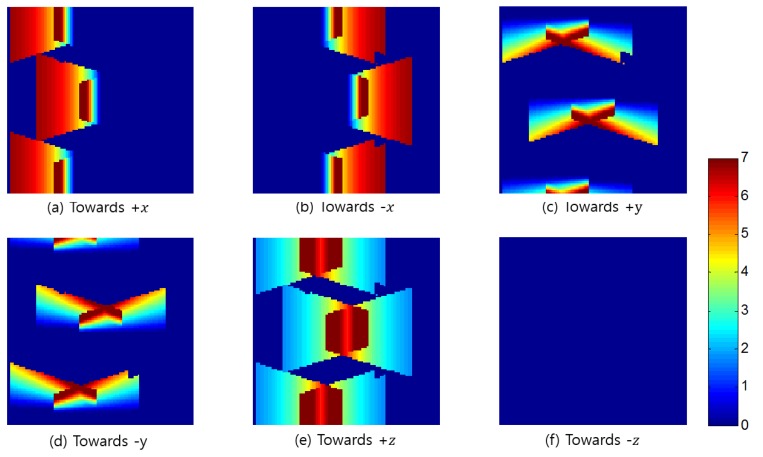
Surveillance resolutions according to the orientation (after adding a camera); (**a**) the resolutions with the +x direction; (**b**) the resolutions with the −x direction; (**c**) the resolutions with the +y direction; (**d**) the resolutions with the –y direction; the resolutions with the −x direction; (**e**) the resolutions with the +z direction; (**f**) the resolutions with the +z direction.

Finally, Figure 12 displays the surveillance resolutions at the most probable orientations at the height of 1.6 m. From this, we can observe that, even though the location of the object is identical, the surveillance resolution significantly differs between the orientations. In the case where the object is facing in the –z direction, as illustrated in Figure 12f, the object does not appear in the CCTV image, even with the additional camera. This implies that, when a person intentionally faces the ground as they travel, their facial features cannot be observed from the CCTV system. To solve this problem, additional cameras facing in an upward direction should be installed at a lower height range of 0 cm to 50 cm, as needed. After installing the additional cameras, we can enhance the surveillance coverage in the area three dimensionally and omni-directionally.

#### 3.2.3. Path Analysis

**Figure 13:**
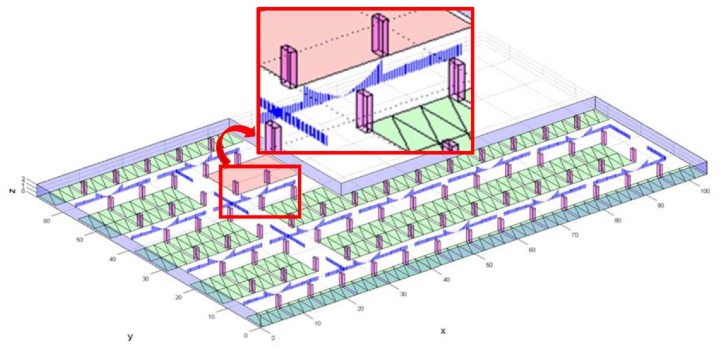
Surveillance resolution at the sampled locations, when the orientation is the one direction where the maximum resolution is attained.

The red line in Figure 4 represents the moving paths for vehicles in the parking lot, where careful surveillance is required. We sampled the paths with an interval of 0.1 m and 0.5 m in horizontal and vertical directions, respectively. At each location, we selected six orientations with a 90° interval. We then computed the surveillance resolution at each sampled location and orientation. We presented the surveillance resolutions of the sampled locations, when the orientation is the one direction where the maximum resolution is attained, using vertical bars, as shown in Figure 13, where the length of a vertical blue bar indicates the magnitude of the surveillance resolution. As shown in Table 2, the surveillance coverage index with the thresholds of 0 px/cm and 1 px/cm are 87% and 83%, respectively.

**Table 2:** Surveillance Coverage Index (SCI) along a certain path.

Threshold (px/cm)	All Directions (E/W/N/S)			Maximum Direction (E/W/N/S)		
	Total	>Threshold	SCI	Total	>Threshold	SCI
0	13864	6908	50%	3466	3016	87%
1	13864	3192	23%	3466	2864	83%

## 4. Conclusions

Although the efficient design and installation of CCTV systems is recognized as important, there is a lack of comprehensive indicators, which are useful to understand quantitatively how well a CCTV system covers a target area while satisfying specific surveillance requirements. In this study, we thus have proposed new indicators, surveillance resolution and coverage index, which allow us to evaluate quantitatively the effectiveness of CCTV systems on the task-specific surveillance. The surveillance resolution, indicating how closely an object can be observed by cameras, is derived from a rigorous projection model from objects to cameras. The derivation reflects the 3D orientation as well as the location of objects and cameras; and it can also be applicable to various kinds of cameras with different projection and distortion characteristics. Based on the surveillance resolution, we defined the surveillance coverage index representing how completely an area is monitored with a certain level of detail. Using these two indicators and the presented derivation associated with them, we established an evaluation process to enable versatile, practical and visual analysis on the CCTV’s surveillance coverage. For example, one can derive the overall surveillance coverage of the entire target area, check whether a specific interesting area (or path) is monitored with a required resolution, and provide various alternatives to improve the current coverage. During these processes, one can easily incorporate the dynamic and static attributes of objects and cameras, for example, the movement of persons or vehicles.

In the near future, we will adapt the proposed evaluation approach to field problems related to the regions of more strict specific surveillance requirements, for example, crime-ridden districts, subway stations, complex malls, casinos, and other places. With the adapted approach, we can assess the current status and provide appropriate solutions. In addition, using the proposed surveillance index as the target values to be optimized, we can provide the optimal position and orientation of the cameras to maximize the performance of a CCTV system according to its surveillance requirements.
